# Alterations in Corneal Sensitivity, Staining and Biomechanics of Alopecia Areata Patients: Novel Findings in a Case-Control Study

**DOI:** 10.3390/jcm13082426

**Published:** 2024-04-21

**Authors:** Barbara Burgos-Blasco, Patricia Burgos-Blasco, Olivia Rodriguez-Quet, Pedro Arriola-Villalobos, Jose Ignacio Fernandez-Vigo, David Saceda-Corralo, Sergio Vaño-Galvan, Julián García-Feijóo

**Affiliations:** 1 Ophthalmology Department, Instituto de Investigación Sanitaria, Hospital Clínico San Carlos (IdISSC), 28040 Madrid, Spain; 2 Trichology Unit, Dermatology Department, Instituto Ramón y Cajal de Investigación Sanitaria—IRYCIS, Ramon y Cajal Hospital, 28034 Madrid, Spain; 3 Departamento de Inmunología, Oftalmología y ORL, Facultad de Medicina, Universidad Complutense de Madrid, 28040 Madrid, Spain; 4 Grupo Pedro Jaen Clinic, 28002 Madrid, Spain

**Keywords:** topography, biomechanical, alopecia areata, ocular, cornea, keratoconus

## Abstract

**Background**: A higher prevalence of ophthalmological alterations in systemic inflammatory diseases has been demonstrated. **Objectives**: Our objectives were to determine anterior segment findings and corneal properties in alopecia areata (AA). **Methods**: This is a case-control study. Severe AA patients (Severity of Alopecia Tool > 50%) and non-AA subjects underwent a general ophthalmological examination, a Pentacam and Corvis scheimpflug technology examination (Oculus Optikgeräte GmbH, Wetzlar, Germany). Visual acuity, refractive error, corneal aesthesiometry, and biomechanical and topographic variables were registered. **Results**: In total, 25 AA patients (50 eyes; 50.6 ± 8.1 years) and 29 controls (58 eyes; 49.4 ± 8.6 years) were included. AA patients had decreased corneal sensitivity, more corneal staining, and a more advanced cataract (*p* ≤ 0.004). The anterior topographic flat meridian, mean anterior keratometry, and maximum keratometric point were increased in AA (*p* ≤ 0.040), while pachymetry values were thinner (*p* ≤ 0.001). Keratoconus index and Belin/Ambrosio-enhanced ectasia total deviation display were increased (*p* ≤ 0.007). Two eyes with a topographic diagnosis of keratoconus and four eyes with subclinical keratoconus were detected in AA. Applanation lengths were smaller in AA (*p* ≤ 0.029). The Corvis Biomechanical Index was increased in AA (*p* = 0.022). **Conclusions**: AA patients have reduced corneal sensitivity and increased corneal staining. Topographic and biomechanical parameters are altered, and there could be a higher risk of keratoconus, thus possibly requiring routine ophthalmological examination.

## 1. Introduction

Alopecia areata (AA) is a common immune-mediated form of non-scarring hair loss of the scalp and other hair-bearing areas of the body. It is the second-most frequent non-scarring alopecia, affecting up to 2% of the global population, as documented by several large epidemiological studies. It can affect all ages, but the prevalence appears to be higher in children compared to adults. A greater incidence has been reported in females than males, especially in patients with late-onset disease, defined as an age greater than 50 years. However, the reported prevalence, age of onset, and history and concurrent diseases can vary widely.

There are multiple different patterns of presentation of hair loss in AA. The most common is a sudden onset of focal, well-circumscribed patches of hair loss on the scalp that are not associated with signs of significant inflammation or scarring [1,2]. The disease may progress to include all scalp hairs (alopecia totalis) or all body hairs (alopecia universalis) [3,4]. The diagnosis of alopecia areata is usually clinical, and further tests are usually not needed. Nonetheless, a number of tools, such as trichoscopy or histopathology, can further validate the diagnosis. Trichoscopy is a simple, rapid technique that reduces the need for invasive procedures and can also help with monitoring treatment response. The most common trichoscopic findings are yellow dots, black dots, exclamation mark hairs, short vellus hairs, and coudability hairs.

Alopecia areata is conventionally classified as patchy, alopecia totalis, and alopecia universalis. A more detailed classification should include the disease duration and the extent of hair loss. Multiple assessment tools exist for the objective scoring of alopecia. The Severity of Alopecia Tool (SALT) Score is commonly used [5]. The tool involves splitting the scalp into four quadrants and summing the percentage of the scalp area devoid of terminal hairs in each quadrant and then the whole scalp to provide the total area affected. It does not account for the loss of facial hair (eyelashes, eyebrows, beard) or body hair. Other scoring systems have been proposed for the assessment of the severity of hair loss in eyelashes, eyebrows, and nail findings based on the qualitative analysis of interviews with expert dermatologists and patients.

Although the skin is the most affected organ, alopecia areata is a systemic inflammatory disease characterized by the interaction of T lymphocytes with follicular antigens. The hair follicle’s immune privilege is disrupted, and inflammatory immune cells lead to dystrophic hair follicle cycling with premature entry into the telogen phase. In addition, AA is associated with an increased overall risk of other autoimmune disorders, such as thyroid disease, psoriasis, and vitiligo [3].

Studies have reported ocular abnormalities in AA patients, although these are mainly retinal and lenticular [6]. Multiple authors have documented an increase in cataract prevalence among AA patients, probably related to corticosteroid therapy [7,8,9]. However, the common embryonic origin of the skin and the lens from the ectoderm could also explain this association [9]. Moreover, a decrease in corneal sensitivity has been classically reported, although it has not been thoroughly documented [10].

Corneal parameters have not yet been investigated in AA. Other autoimmune diseases like hyperthyroidism and rheumatoid arthritis have been linked to corneal alterations, including keratoconus [11,12,13]. To the best of our knowledge, there is no research reporting corneal topographic and biomechanical findings in patients with AA in the literature. Therefore, the aim of this study was to investigate corneal topography parameters as well as biomechanics in patients with alopecia areata compared to non-AA individuals.

## 2. Methods Section

### 2.1. Design of the Study

This is a case–control study including AA patients and non-AA individuals that was conducted at the Hospital Ramón y Cajal and the Hospital Clinico San Carlos in Madrid, Spain. Written informed consent was obtained, and the protocol of the study was approved by both hospitals’ Ethics Committees (21/216-E approved 31 March 2021). The study was performed in accordance with the tenets of the Declaration of Helsinki, and the STROBE guidelines were followed.

### 2.2. Participants and Recruitment

Participants with severe AA were recruited consecutively from the Trichology Unit of the Hospital Ramón y Cajal by the dermatologists. Inclusion criteria were more than 18 years of age, clinical diagnosis of severe AA (extensive multifocal AA, total or universal, with >50% involvement of the scalp according to the SALT scale) by a dermatologist, no systemic treatment for AA at least in the previous 4 weeks for standard treatment and 12 weeks for biological or anti-JAK drugs.

For the control group, non-AA subjects were consecutively selected from the general ophthalmology of the Hospital Clinico San Carlos. Inclusion criteria for the control group were over 18 years of age, informed consent, and a routine visit to the Ophthalmology Department. As exclusion criteria, we used the diagnosis of AA by a dermatologist, a family history of AA of any severity, and a previous diagnosis of other chronic inflammatory diseases. Patients with a previous diagnosis of ophthalmologic conditions were excluded from both groups.

### 2.3. Outcomes and Assessments

At the Ophthalmology Department of the Hospital Clinico San Carlos, all participants underwent an ophthalmic examination, including slit lamp biomicroscopy, to confirm the inclusion and exclusion criteria. All the ophthalmological exams were performed in a single visit.

First, the following characteristics were noted from the medical history: age, sex, presence of systemic diseases (atopia, arterial hypertension, diabetes, dyslipidemia, hypothyroidism, asthma, osteoporosis, and another diagnosis), previous ocular surgeries, and previous ocular diseases. In the AA group, SALT, age at diagnosis, and time since diagnosis were recorded. Then, best-corrected visual acuity (BCVA) with the logMAR scale, refractive error, axial length, and corneal aesthesiometry were measured. For corneal sensitivity, a Cochet–Bonnet esthesiometer was used. An anterior segment slit lamp examination was also performed, and the following ordinal variables were registered: conjunctival hyperemia (McMonnies classification), corneal staining (Oxford classification), cataract (Lens Opacities Classification System version III), and other findings. Intraocular pressure was also measured.

Corneal topography was evaluated using the Pentacam HR (Oculus Optikgeräte GmbH, Wetzlar, Germany). This device uses a monochromatic blue light-emitting diode with a wavelength of 475 nm and a Scheimpflug camera, which rotates around the corneal axis. Only eyes with good-quality images are included. Of each examination, the following quantitative variables were collected: anterior topographic flat meridian (K1), anterior topographic steepest meridian (K2), mean anterior keratometry (anterior Km), mean posterior keratometry (posterior Km), maximum keratometric point (Kmax), surface variance index (ISV), vertical asymmetry index (IVA), keratoconus index (KI), central keratoconus index (CKI), highest asymmetry index (IHA), highest decentration index (IHD) and posterior elevation. Furthermore, the Pentacam provides a keratoconus scale (TKC) between absent and grades 0.5 to 4 according to the KC classification system of Amsler–Krumeich (ordinal variable). From the improved ectasia screen of Belin/Ambrosio, the following quantitative parameters were included: Ambrosio-related maximum thickness (ART-Max) and the Belin/Ambrosio deviation index (BAD-D). Also, the thinnest corneal thickness and apex corneal thickness were registered.

Biomechanical parameters were obtained using Corvis scheimpflug technology (Oculus Optikgeräte GmbH, Wetzlar, Germany). Corvis measures the biomechanical response of the cornea to a defined air pulse, characterizing the moment of the first and second applanations and highest concavity events. The length of the flattened segment and the corneal movement velocity during applanation at the moment of both the first and second applanations were included. Also, the highest concavity deformation amplitude, the distance between the bending points of the cornea (highest concavity peak distance), and the central concave radius of curvature at the point of highest concavity were noted. Lastly, a combined biomechanical index called the Corvis Biomechanical Index (CBI) based on the corneal thickness profile and deformation parameters was registered. All the biomechanical variables were evaluated quantitatively.

### 2.4. Statistical Analysis

Statistical analysis was performed using SPSS version 25.0 (SPSS, Chicago, IL, USA). Quantitative variables are represented by their mean, along with their standard deviation (SD) and range, while qualitative variables are shown as proportions. Differences between the groups and the measurements were investigated using the Mann–Whitney U test (nonnormality was assumed). *p* < 0.05 was considered statistically significant.

## 3. Results

The study population comprised 25 patients (50 eyes) with AA and 29 controls (58 eyes). An AA patient was excluded from a previous diagnosis of keratoconus, which had required intrastromal ring segment implantation. No controls were excluded for this motive.

The mean age of AA and controls was 50.6 ± 8.1 and 49.4 ± 8.6 (*p* = 0.888), respectively. In total, 18 of the AA patients (72%) were women, as well as 22 (76%) of the non-AA controls. The time since AA diagnosis was 26.3 ± 13.5 years (range 6–46). Of the AA patients, 21 (84%) had 100% SALT, 2 (8%) had 95%, and 2 (8%) had 80% SALT. Three patients (12%) had atopia, 5 (20%) had arterial hypertension, none had diabetes, none had dyslipidemia, 10 (40%) had hypothyroidism, 1 (4%) had asthma, 2 (8%) had osteoporosis and 2 patients (8%) had a diagnosis of anxiety.

The BCVA of AA patients was 0.05 ± 0.1 logMAR, which was significantly worse than that of the control group (−0.05 ± 0.12 logMAR; *p* < 0.001). Upon examination, AA patients had a decreased corneal sensitivity (*p* < 0.001), more corneal staining (*p* = 0.004), and a more advanced cataract (*p* < 0.001; Table 1). All patients with cataracts had nuclear cataracts.

**Figure 1:**
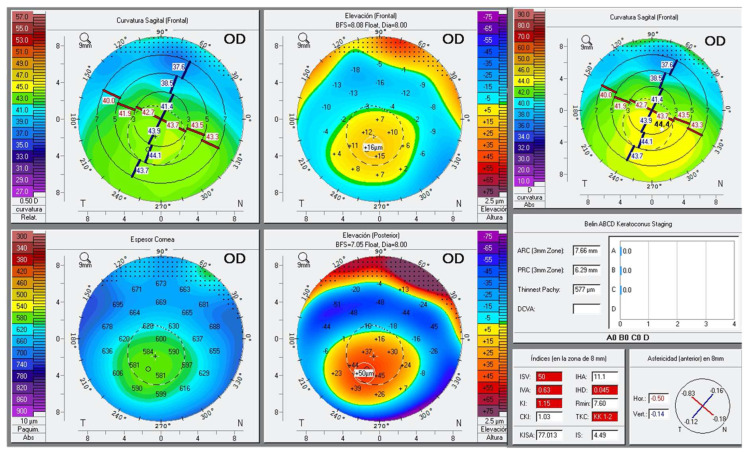
Corneal topography of one of the patients with keratoconus.

As for topographical parameters (Table 2), K1, anterior Km, and Kmax were significantly increased in the AA group (*p* < 0.05). Although values were higher in AA patients, differences in K2, posterior elevation, or posterior Km were not statistically significant. Thinner pachymetry values were detected in AA patients (*p* ≤ 0.001). KI showed a significant increase among AA patients (*p* = 0.007), as well as BAD-D (1.3 ± 0.7 vs. 0.8 ± 0.6; *p* = 0.026). ART-max showed a significant decrease in AA patients. In addition, two eyes (4%) with a topographic diagnosis of keratoconus and another four eyes (8%) with subclinical keratoconus were detected in the AA group (Figure 1). No cases of clinical or subclinical keratoconus were noted among the controls.

Regarding the biomechanical analysis of the cornea, applanation 1 and 2 velocities were similar in both groups, while applanation lengths were significantly smaller in AA patients (Table 3). CBI was increased in AA patients (0.37 ± 0.25 vs. 0.25 ± 0.25; *p* = 0.022).

## 4. Discussion

AA is a systemic inflammatory disease in which multiple ocular alterations have been described [6]. In this study, the anterior segment was thoroughly investigated, revealing decreased corneal sensitivity, more corneal staining, and a more advanced cataract in AA. Topographic and biomechanical parameters showed differences between groups, and three patients were diagnosed with corneal ectasia.

In AA, an increase in cataract prevalence has been reported by multiple groups, and some have suggested that cataract formation might reflect ectodermal reactivity or be related to associations of atopic dermatitis and vitiligo [9,14,15,16,17]. This finding is consistent with a more common use of steroids and an increase in oxidative stress in AA [18]. In contrast, Orecchia et al. [6] stated that lens opacities do not have any significant clinical relevance in AA, and other authors have not detected differences in visual acuity [14]. In our series, cataracts were probably the reason for a difference in visual acuity, which, although statistically significant, was small. The cataracts noted were nuclear and not posterior subcapsular, which would be more consistent with previous steroid use.

As for the ocular surface and the prevalence of dry eye disease among AA patients, some authors have reported a higher Ocular Surface Disease Index (OSDI) and corneal staining stage scores, along with a lower tear break-up time compared to controls [16,19]. Ergin et al. also noted papillary hypertrophy to be more prevalent in the patient group, while Oltulu et al. observed that patients with AA had more squamous metaplasia transformation in the conjunctival epithelium. However, no differences in the Schirmer test results were reported by most groups [14,16,19]. Nevertheless, an altered tear function and quality in AA has been suggested, which is further supported by our results. Tear stability seems to be the main issue, possibly secondary to the loss of goblet cells due to inflammation [19]. The association between dry eye disease and AA could also be explained by a common T-cell-mediated autoimmunity pathogenesis.

Reduced corneal sensitivity has been established as part of the signs of AA patients since its first cases were reported [10]. This case series is the first to clearly document a reduced corneal sensitivity, although its clinical relevance and causes are still to be investigated. Corneal sensitivity is reduced in dry eye disease, specifically in the aqueous tear deficiency subset. Another possibility is that chronic inflammation induced by tear dysfunction and the disease itself may contribute to corneal nerve degeneration and, thus, reduce corneal sensitivity [20].

Furthermore, corneal alterations have already been described in AA patients, and several cases of keratoconus have been reported. However, cases reported include another risk factor for corneal ectasia besides AA. For example, a ten-year-old patient with atopic keratoconjunctivitis (very symptomatic and intense eye rubbing), Hashimoto thyroiditis, and alopecia areata also presented with keratoconus [21]. Autoimmune diseases, keratoconjunctivitis, and eye rubbing are all risk factors for keratoconus. In our series, two eyes were diagnosed with topographic keratoconus and another four eyes with subclinical keratoconus. Interestingly, one AA patient was excluded due to keratoconus with the previous implantation of intracorneal ring segments.

Our analysis of corneal parameters shows that AA patients have altered topography parameters. This is further supported by the topographic index, such as BAD-D, KI, and ART-max. Only Esmer et al. [14] evaluated further corneal characteristics in AA besides the slit lamp examination, and no differences in the keratometric measurement were detected, although these were analyzed using autokeratorefractometry, thus not allowing for detailed evaluation. In that study, no differences in central corneal thickness were noted, but patients were young, and the time since diagnosis, although not described, was probably lower than in the present study. Although differences in several parameters, which are sometimes observed in patients with corneal ectasia, were noted, mean values in AA patients were still within the normal range.

Biomechanical analysis of the cornea showed smaller applanation lengths in AA patients and an increase in CBI. This is in agreement with the differences noted between corneal ectasia and non-AA patients, although Elham et al. also noted differences in times and velocities [22]. The other biomechanical variables share a trend with these results, although they did not reach statistical significance. The tomographic biomechanical index is considered the most sensitive index to verify mild ectasia and could be useful [23].

Multiple mechanisms could explain these corneal topographic and biomechanical changes. Severe AA patients lack eyelashes, which have a protective role. In addition, atopy is twice as common in AA, which could promote eye rubbing, which is a main risk factor for keratoconus. Our patients did not report intense eye rubbing, although four patients referred to occasional eye rubbing and used tear substitutes frequently. Strong associations between keratoconus and multiple allergic and autoimmune diseases have been documented, including rheumatoid arthritis, ulcerative colitis, and Hashimoto thyroiditis, among others [11]. This supports the key role of the immune system in the pathogenesis of keratoconus and could be of relevance in the development of keratoconus in AA.

Interestingly, corneal alterations and keratoconus have also been reported in psoriasis. In this regard, Akcam et al. [24] performed corneal topography in patients with psoriasis controls, finding a possible association with keratoconus. The mean index of vertical asymmetry value was significantly increased in psoriatic patients. More interestingly, 26 eyes were considered keratoconus suspects, and two of them were diagnosed with definite keratoconus. A positive correlation was found among topometric parameters, especially between the duration of the disease and PASI score, while a negative correlation was discovered between topometric parameters and the early beginning of psoriasis. Other groups have described alterations in corneal biomechanics, although another device was employed [25,26]. Therefore, the common inflammatory basis between AA and psoriasis supports inflammation as a key phenomenon in the pathogenesis of keratoconus in these patients.

### 4.1. Limitations

Some limitations should be acknowledged. Firstly, the association found does not prove causality, although these results support the development of a referral protocol from the Dermatology Department to ophthalmologists, and the present findings strongly suggest a higher incidence of corneal ectasia in AA. Secondly, a prospective design should be considered to further investigate changes in corneal parameters over time. Also, the sample included in this study is small, but the severity of the patients recruited made it difficult. Given that the patients included presented with severe AA, the possible associations or changes in milder forms of the disease are still unknown.

### 4.2. Future Research

In order to prove the associations evaluated in this study, a specific population-based study would be necessary. Secondly, a prospective design should be considered to further investigate changes in corneal parameters over time. Also, a larger sample size would be preferred, preferably with different severities of the disease.

**Figure 2:**
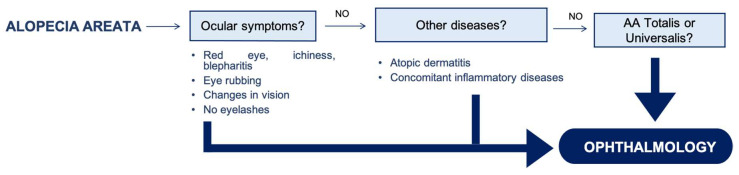
Proposed algorithm for the referral of alopecia areata patients to the Ophthalmology Department.

Nevertheless, the current study strongly suggests a higher incidence of anterior segment alterations in AA patients, which include decreased corneal sensitivity, more corneal staining, a more advanced cataract, and topographic and biomechanical changes. Based on these findings, we propose an algorithm to decide which AA patients should be referred to the Ophthalmology Department (Figure 2). This is based on identifying those patients with a higher risk of corneal alterations, as the initial stages of keratoconus may go unnoticed due to a lack of symptoms. Hence, AA patients with ocular symptoms (itchiness, red eye, blepharitis, eye rubbing, or changes in vision), no eyelashes, other diseases (atopic dermatitis or concomitant inflammatory diseases), as well as severe AA (totalis or universalis), may benefit from an ophthalmological exam.

## 5. Conclusions

In summary, this is the first study to thoroughly evaluate corneal parameters in AA, depicting how topographic and biomechanical parameters are altered in AA and how patients may present with a higher incidence of corneal ectasia. Our findings suggest that patients with severe AA should undergo a complete ophthalmological examination and a prolonged follow-up to allow for early diagnosis of ocular diseases and prevent future ocular morbidity.

## Figures and Tables

**Table 1:** Ophthalmological examination results in alopecia areata patients compared to non-AA controls.

Characteristics	Non-AA Controls				Alopecia Areata Patients				*p*
	Mean	SD	Range		Mean	SD	Range		
Visual acuity	−0.05	0.12	0.70	−0.12	0.05	0.10	0.30	−0.12	<0.001 *
Cochet–Bonnet aesthesiometry	5.47	0.74	4.00	6.00	3.73	1.32	0.50	6.00	<0.001 *
Axial length	23.70	1.25	21.31	27.24	23.21	0.83	21.24	24.93	0.052
Refractive error	−0.56	2.64	−9.75	4.75	−1.16	1.66	−5.75	3.00	0.027 *
Intraocular pressure	13.74	2.90	10.00	18.00	13.91	1.72	9.00	16.00	0.618
Conjunctival hyperemia	0.13	0.37	0.00	2.00	0.04	0.21	0.00	1.00	0.096
Corneal staining	0.44	0.66	0.00	2.00	0.11	0.30	0.00	1.00	0.004 *
Cataract	2.15	0.72	0.00	3.00	1.00	1.40	0.00	4.00	<0.001 *

SD: standard deviation. * indicates *p* < 0.05.

**Table 2:** Topographic analysis in alopecia areata patients and non-AA controls.

Topographic Variables	Non-AA Controls				Alopecia Areata Patients				*p*
	Mean	SD	Range		Mean	SD	Range		
K1	42.79	1.57	39.70	46.50	43.44	1.07	41.50	45.50	0.019 *
K2	43.83	1.67	40.60	48.60	44.30	1.04	42.30	46.60	0.090
Anterior Km	43.31	1.59	40.20	47.40	43.86	1.00	41.90	46.00	0.033 *
Posterior Km	−6.15	0.78	−6.70	−0.62	−6.30	0.17	−6.70	−6.00	0.145
Kmax	44.55	1.83	40.90	49.60	45.11	1.10	42.70	47.10	0.040 *
ISV	17.10	5.84	7.00	39.00	18.54	7.39	7.00	50.00	0.357
IVA	0.13	0.05	0.04	0.30	0.15	0.09	0.04	0.63	0.308
KI	1.01	0.02	0.97	1.05	1.02	0.03	0.94	1.15	0.007 *
CKI	1.00	0.01	0.99	1.02	1.00	0.01	0.99	1.03	0.409
IHA	5.30	3.85	0.10	15.90	5.95	4.20	0.10	15.70	0.436
IHD	0.01	0.01	0.00	0.03	0.02	0.03	0.00	0.18	0.795
Posterior elevation	8.47	4.15	2.00	25.00	10.32	5.86	2.00	35.00	0.054
ART-max	492.21	105.63	244.00	724.00	452.44	99.49	242.00	728.00	0.026 *
BAD-D	0.77	0.61	−0.44	2.12	1.25	0.68	−0.41	3.01	<0.001 *
Apex corneal thickness	566.60	30.85	504.00	637.00	546.74	25.78	499.00	607.00	0.001 *
Thinnest corneal thickness	559.52	36.37	402.00	629.00	540.84	25.19	497.00	603.00	<0.001 *

SD: standard deviation; K1: anterior topographic flat meridian; K2: anterior topographic steepest meridian; Km: mean keratometry; Kmax: maximum keratometric point; ISV: surface variance index; IVA: vertical asymmetry index; KI: keratoconus index; CKI: central keratoconus index; IHA: highest asymmetry index; IHD: highest decentration index; ART-max: Ambrosio-related maximum thickness; BAD-D: Belin/Ambrosio deviation index. * indicates *p* < 0.05.

**Table 3:** Biomechanical analysis in alopecia areata patients and non-AA controls.

Biomechanical Variables	Non-AA Controls				Alopecia Areata Patients				*p*
	Mean	SD	Range		Mean	SD	Range		
Applanation 1 length	2.35	0.33	1.65	3.00	2.19	0.31	1.80	2.75	0.029 *
Applanation 1 velocity	0.14	0.02	0.10	0.18	0.15	0.02	0.09	0.18	0.415
Applanation 2 length	2.01	0.41	0.99	3.16	1.82	0.28	1.04	2.69	0.010 *
Applanation 2 velocity	−0.26	0.04	−0.31	−0.03	−0.26	0.03	−0.29	−0.19	0.885
Peak distance	4.91	0.28	4.20	5.49	4.87	0.26	4.16	5.26	0.458
Concave radius	6.77	0.82	3.81	8.53	6.56	0.82	3.94	9.45	0.078
Deformation amplitude	1.09	0.11	0.86	1.29	1.10	0.11	0.88	1.32	0.414
CBI	0.25	0.25	0.00	0.88	0.37	0.25	0.02	0.87	0.022 *

SD: standard deviation; CBI: corneal biomechanical index. * indicates *p* < 0.05.

## Data Availability

The original contributions presented in the study are included in the article, further inquiries can be directed to the corresponding author.
